# The Effect of Microballoon Volume Fraction on the Elastic and Viscoelastic Properties of Hollow Microballoon-Filled Epoxy Composites

**DOI:** 10.3390/ma16247554

**Published:** 2023-12-07

**Authors:** Rojer Chitrakar, Md Sakhawat Hossain, Sabrina Nilufar

**Affiliations:** School of Mechanical, Aerospace, and Materials Engineering, Southern Illinois University Carbondale, Carbondale, IL 62901, USA

**Keywords:** syntactic foam, microballoon, finite element analysis (FEA), Random Sequential Adsorption (RSA), Dynamic Mechanical Analysis (DMA), Representative Elementary Volume (REV)

## Abstract

This paper reports the study of hollow microballoon-filled epoxy composites also known as syntactic foams with various volume fractions of microballoons. Different mechanical and thermomechanical investigations were carried out to study the elastic and viscoelastic behavior of these foams. The density, void content, and microstructure of these materials were also studied for better characterization. In addition to the experimental testing, a representative 3D model of these syntactic foams was developed to further investigate their elastic behavior. The results indicate that changes in the volume percentage of the microballoons had a substantial impact on the elastic and viscoelastic behavior of these foams. These results will help in designing and optimizing custom-tailored syntactic foams for different engineering applications.

## 1. Introduction

Syntactic foams are hollow particle-filled composites that are widely used in areas that require high strength while maintaining low weight and density. These foams are highly tailorable materials whose properties can be altered during the manufacturing process by changing various parameters such as matrix and microballoon material type, size, and distribution, as well as the volume fraction and wall thickness of microballoons. The word ‘syntactic’ is derived from the Greek word ‘syntaktikoÂs’, meaning ‘orderly disposed system’. Therefore, syntactic foams are composites made by dispersing hollow microspheres/microballoons into matrix material [1,2,3]. The matrix material can be metal, polymer, or ceramic and the microspheres can be made up of various materials like glass, ceramic, or cenospheres [4,5,6,7]. The introduction of the microspheres helps in enhancing the properties of the matrix material like its moisture absorption, buoyancy, specific strength, damage tolerance, thermal tolerance, and energy dissipation capacity [8,9]. Another advantage of syntactic foams is that they are highly tailorable, giving greater flexibility during the manufacturing process [10,11,12]. Wide varieties of properties for numerous applications can be obtained by independently altering various parameters like matrix and microballoon material, type, size, and distribution, as well as the volume fraction and wall thickness in syntactic foams [10,13]. These variations give the syntactic foam lots of advantageous properties over conventional materials [14,15,16] which make it a popular choice as a core material in sandwich structures in numerous engineering applications. Syntactic foams were initially used in marine applications due to their light weight, buoyancy, low moisture absorption, and high compressive strength. However, with a better understanding of the material and its properties, it has now been used in various applications where lightweight but strong materials are needed such as aerospace, automotive, civil, consumer goods, and even petrochemical industries [8,11,17,18]. Additionally, the potential practical applications of syntactic foams include pipe insulation, spacecraft, boat hulls, underwater vehicles, and soccer balls.

There have been various studies in the past that have investigated the properties of syntactic foam both through experimental and numerical approaches. The effect of microballoon volume fraction on the elastic behavior and the fracture mechanics of the syntactic foam in compression loading was studied [19]. It was found that Young’s modulus and the critical strain of the syntactic foam increased with the increase in the volume fraction of the microballoons. It was also demonstrated that cracks propagated longitudinally in syntactic foams with a low volume fraction of microballoons. On the contrary, for foams with a higher volume fraction of microballoons, cracks propagated diagonally. Similarly, the effect of the microballoon density on the tensile modulus and the tensile strength of the syntactic foam were also evaluated in the past [20]. It was concluded that the tensile modulus of syntactic foam not only depends on the volume fraction of the microballoons but also the density of the microballoons being used. As the volume fraction of the microballoons decreased, the modulus of syntactic foam containing low-density microballoons increased. However, for higher density microballoons, there was no significant effect of the volume fraction on its modulus. It was also discovered that the tensile strength increased with the density and decreased with an increase in the volume fraction of the microballoons with the same density. Furthermore, other studies also found that, for the same volume fraction, if the density of the microballoon is increased, the density of the overall syntactic foam is also increased [21]. There are numerous studies to analyze the changes in the mechanical behavior of syntactic foam with varying diameters of hollow microballoons. The effect of wall thickness to the radius ratio of the microballoons in the mechanical behavior of the foam was studied and it was found that the microballoons with a smaller wall thickness to diameter ratio are more resistant to crack propagation [22]. The effect of the true particle density of the microballoons and their volume fraction on the thermophysical properties of the syntactic foam was also studied. It was reported that, at room temperature, the storage modulus of the syntactic foam increased with the increased wall thickness of the microballoons [23], whereas it decreased with the increase in its volume fractions [23]. In a similar study, it was reported that the presence of glass microballoons helps increase the mechanical properties of syntactic foam at a temperature beyond the glass transition temperature [24]. Although there have been a lot of studies in this field, considering their customizability, several parameters can be tweaked to enhance and change their properties. Therefore, more studies and testing are necessary for a better understanding and characterization of these composites.

One of the key methods to control the properties of syntactic foam is to alter its constituents’ volume fractions. By changing the microballoon volume fraction, we can change bulk properties including compressive [21,25], tensile [26,27], and flexural properties [7,28,29]. Moreover, the unlimited choice of constituting materials for syntactic foam makes it difficult to find studies using the same constituents. Thus, it is helpful to utilize numerical modeling and simulation to predict the behavior of the foam [18,30,31]. Numerical modeling and finite element analysis (FEA) have been very effective tools in understanding and predicting the properties of various linear and non-linear materials [32,33,34]. The most common numerical models used to study the overall properties of the composite materials are the unit cell method and Representative Elementary Volume (REV) [19,35,36,37,38]. Moreover, during numerical modeling, the microballoons should be randomly distributed to accurately imitate the physical foam. This random distribution of the microballoons can be achieved using different algorithms like Random Sequential Adsorption (RSA) and the Lubachevsky–Stillinger algorithm (LS) [19,27]. RSA is limited to the lower volume of the spheres only (<30%), whereas the LS algorithm is very capable of placing a high volume fraction of microballoons close to the theoretical packing fraction limit [39]. Thus, the obtained models were used in various computational software like ANSYS 2010 [25,32,40] and ABAQUS 6.12 [27,41] to simulate mechanical testing and to predict the behavior and properties of the foams. All these aforementioned studies used different combinations of resins and microballoons such as Urethane-modified oxazolidone-epoxy resin, Novola, DGEBA resin, DER 332 epoxy resin, vinyl ester resin, E51 resin with borosilicate, S22, K1, K15, K25, and K46 microballoons to study the behavior of syntactic foams. However, the specific combination of DER 332 epoxy resin and K25 glass microballoon has never been investigated before.

This work aims to study the effect of the microballoon volume fraction on the elastic and viscoelastic properties of syntactic foams fabricated with DER 332 epoxy resin and K25 glass microballoon. Epoxy resin is a versatile and highly adhesive material with exceptional bonding properties, is resistant to chemicals and moistures, and has the ability to withstand moderate temperatures. Moreover, the reason behind choosing the epoxy-based resin is that it is more energy-absorbent than other syntactic foams [1]. Uniaxial compression testing was carried out with syntactic foams of various volume fractions to examine their mechanical properties. Additionally, to comprehend the effect of the microballoon volume percentage on its viscoelastic behavior, the Dynamic Mechanical Analysis (DMA) of the syntactic foams was also conducted. Finally, by randomly placing the microballoons in the Representative Elementary Volume (REV), a 3D model of the syntactic foams with various volume fractions (0.2–0.5) was created. Finite element analysis was carried out to study the behavior of the foam in the elastic region and compared it with experimental results. These FEA models and the experimental observation will help to better characterize and understand the thermomechanical properties of syntactic foams.

## 2. Materials and Methods

### 2.1. Materials and Microstructure

**Figure 1:**
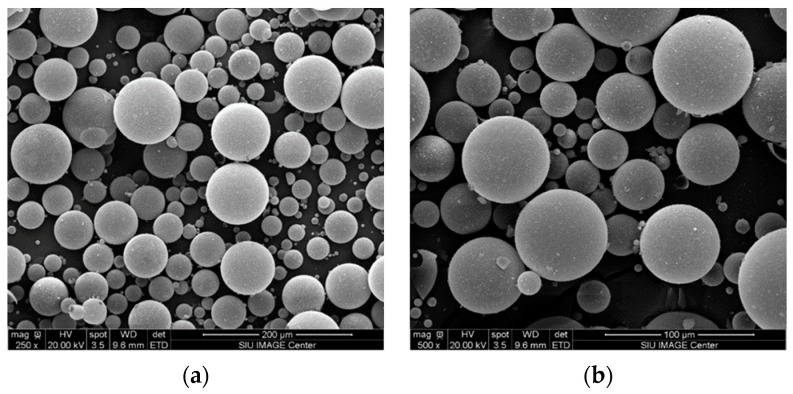

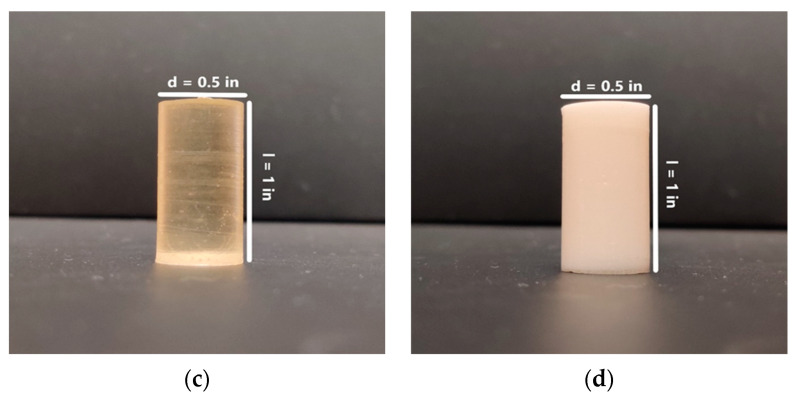
(**a**) SEM images of K25 microballoons showing its microstructure at 250× magnification, (**b**) SEM images of K25 microballoons at 500× magnification, (**c**) neat resin compression sample, and (**d**) compression sample made up of 20% K25 microballoons.

In this study, DER332 epoxy resin (density 1.160 g/cc) and DEH 24 hardener (density 0.981 g/cc) from the DOW Chemical Company were used as the matrix material and curing agent, respectively. In total, 3M Scotchlite™ glass microballoon K25 (density 0.25 g/cc) (Saint Paul, MN, USA) was used as filler material. Figure 1a,b are scanning electron microscopy (SEM) images of the K25 microballoons at 250× and 500× magnifications, respectively. These micrographs provided accurate information on the size distribution of the microballoons. The average size of the microballoons was found to be around 55–60 μm, as seen in Figure 1a,b. These figures confirmed the data provided by the material data sheet from the manufacturer.

Syntactic foams of various volume fractions (0.2, 0.3, 0.4, and 0.5) were fabricated by using the stir casting method. The resin-to-hardener volume ratio was maintained at 14:1 as recommended by the manufacturer. The resin was initially kept in an oven at 50 °C for a few minutes to reduce its viscosity from 4000 cps to 360 cps which aided in the better wetting and mixing of the microballoons and helped in pouring the mixture into the mold [42,43]. The glass microballoons were added in parts and mixed slowly to avoid agglomeration, minimize damage to the glass microballoons, and avoid void formation [15,19]. After the mixture was uniform, it was kept in a vacuum for 20 min to remove any air bubbles trapped in the mixture. The bubble-free mixture was poured into molds coated with a releasing agent (Loctite Frekote 770-NC (Rocky Hill, CT, USA) semipermanent release agent) and left to set at room temperature for 24 h. After the specimen was set, it was post-cured at 100 °C for 3 h [1,2,43]. The cured syntactic foams were then cut and sanded into dimensions specified by ASTM standards for compression and DMA testing to prepare them for mechanical testing.

### 2.2. Property Evaluation

The bulk densities of the foams were calculated from the cuboid samples of dimension length (l) = 1 in, width (b) = 0.5 in, and height (h) = 0.5 in. These densities were then used to determine the void content of the specimen by comparing them to the theoretical densities of the samples that were determined by applying the rule of mixtures [14]. The void volume of syntactic foam was calculated using:

$$V_{void}=V-\left(\frac{V_{mb}*W}{\rho_{mb}}+\frac{V_{m}*W}{\rho_{m}}\right)$$

where *W* is the weight of the syntactic foam specimen; *V_void_* and *V* represent the volume of the void and the syntactic foam; *V_mb_* and *V_m_* represent the volume fractions of the microballoon and matrix; and ρ*_m_* and ρ*_mb_* represent the densities of matrix and microballoon, respectively.

For compression testing, foam samples of diameter, d = 0.5 in and length, l = 1 in were prepared (as shown in Figure 1c,d). The compression samples were prepared according to the ASTM-D695-15 [44] standard having the aspect ratio of l/d = 2.0. The uniaxial compression test was carried out in a Universal Testing Machine by MTS (Eden Prairie, MN, USA), which can apply loads up to 30 KN. The samples were subjected to loading at a constant strain rate of 0.05 mm/min until any major failure occurred.

Several test specimens were fabricated using ASTM D4065-20 [45] standard with various volume fractions of microballoons for the DMA tests. The samples were machined to 13 × 65 × 3 (mm^3^) dimensions and the tests were carried out using a single cantilever beam method on a DMA 850 machine from TA instruments. The temperature was ramped from room temperature to 125 °C at a rate of 5 °C/min, with a frequency of 1 Hz, and an amplitude of 30 μm.

### 2.3. Finite Element Analysis (FEA)

**Figure 2:**
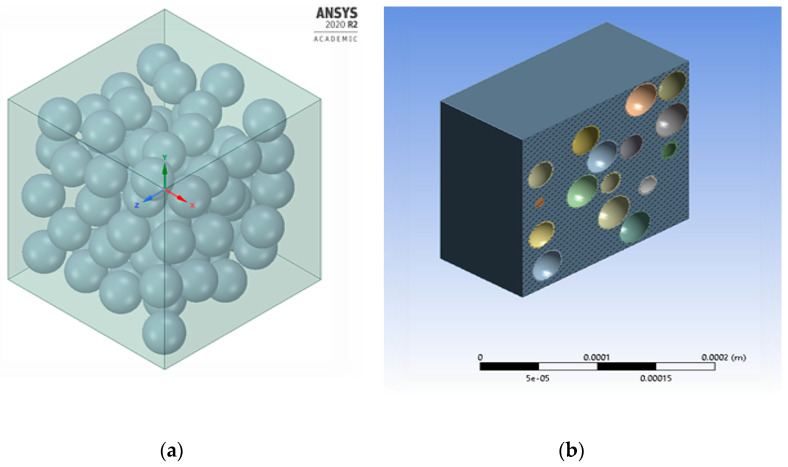
FEA model of a syntactic foam showing (**a**) microballoon distribution in REV and (**b**) microballoon distribution in section view.

**Figure 3:**
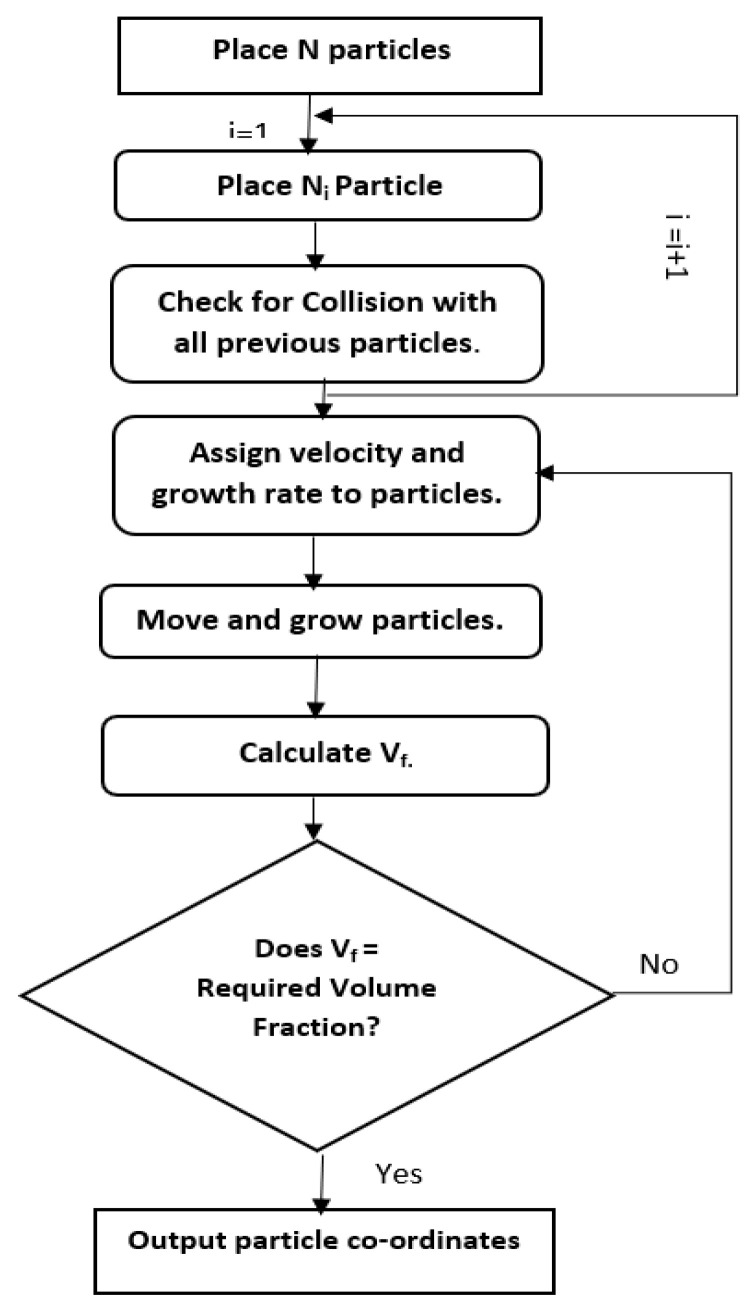
The flowchart of generating the random location of the glass microballoons in REV using LS algorithm.

A 3D model of a cubic Representative Elementary Volume (REV) of dimension 0.2 × 0.2 × 0.2 (mm^3^) was modeled using the ANSYS Workbench 2020 R2 (ANSYS Inc. Canonsburg, PA, USA) to predict the macroscopic elastic behavior and properties of foam as shown in Figure 2. The microballoons were randomly placed within the REV of the syntactic foam using the Lubachevsky–Stillinger (LS) algorithm to achieve the required volume percent. The working principle of the algorithm is shown in the flowchart in Figure 3. The outer diameter of the spheres was assumed to be in a range of 55–60 µm as mentioned in the material data sheet and observed in the SEM images. The theoretical values used in the FE model were Young’s modulus of Egl = 60 GPa for the glass microballoons and Eep = 1.97 GPa for the epoxy resin. The locations of the microballoons were generated such that no overlapping occurred between two adjacent microballoons. The C++ programming language was used to generate the coordinates of these locations. Then, those coordinates were used to create the geometry in the ANSYS Space claim using Python Scripts (as shown in Figure 3). Only the elastic deformation of the REV syntactic foam was carried out in ANSYS to avoid the debonding between the glass microballoons and epoxy matrix [19]. The microballoons and the matrix were subjected to bond constraints in ANSYS and were meshed using tetrahedral elements. The bottom surface of the REV was fixed while the upper surface was subjected to displacement equivalent to a strain rate of the experiment at 0.05 mm/min.

## 3. Results and Discussion

### 3.1. Density and Void Content

The syntactic foams are named SF20, SF30, SF40, and SF50 for convenience, where ‘SF’ is the abbreviation for ‘Syntactic Foam’ and the numeric value corresponds to the volume percentage of the microballoons present in the syntactic foam. The density of SF with various volume percentages of glass microballoon ranging from 20% to 50% is shown in Table 1. The syntactic foams showed a gradual decrease in density with an increase in the volume fraction of microballoons. A 42.63% decrease in the density of foam (SF50) compared to the density of the neat resin (1.145 g/cc) samples was observed. This is due to the addition of the low-density microballoon K25 to a higher density epoxy resin. Moreover, the table also lists the void content in the foam samples. The entrapment of the air during the mixing of the resin and the microballoons results in the formation of voids in the syntactic foams. Although the mixtures are kept in a vacuum to remove these voids, some of those remain entrapped in the samples. These voids can also occur due to the non-uniform distribution of resin and microballoons in the mold. This happens specifically for mixtures containing a higher volume percentage of microballoons. The mixture becomes thicker, and it is harder to pour into the mold. The increased viscosity of the mixture also makes it harder for trapped air to escape during the vacuum operation. Therefore, there is an increase in void content with the increase in the microballoon volume fraction. These voids also result in the lower observed densities of the foams compared to the calculated theoretical densities, as shown in Table 1.

### 3.2. Mechanical Properties

**Figure 4:**
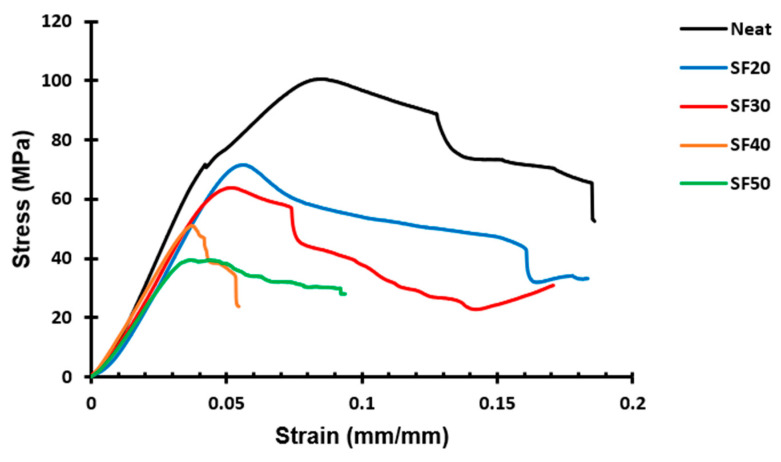
Representative experimental stress–strain curves from the compression testing of syntactic foams with different volume fractions of microballoons.

Figure 4 depicts the typical stress–strain curves for syntactic foams with various glass microballoon volume fractions. These curves can be divided into different regions: the elastic region and the plateau region [19]. According to the graph, the starting point of the fracture is where the stress value reaches its maximum. The initial approximately linear region, before the peak stress value, represents the elastic deformation of the syntactic foams. Hence, it is known as the elastic region. The plateau region occurs after the curve reaches the peak stress value in most of the porous composite. In this region, the glass microballoons start to break under the load and the hollow voids then formed start to be filled by the matrix material and the debris of the microballoons. As a result, syntactic foams begin to yield. However, these curves have a very insignificant plateau region. This absence of the plateau region can be attributed to the low volume fraction and the low density of microballoons added, which does not provide enough reinforcement to the matrix materials and all the failures were due to the fracture of the matrix itself rather than the microballoon. The plateau area corresponds to how much energy the foam can absorb.

**Figure 5:**
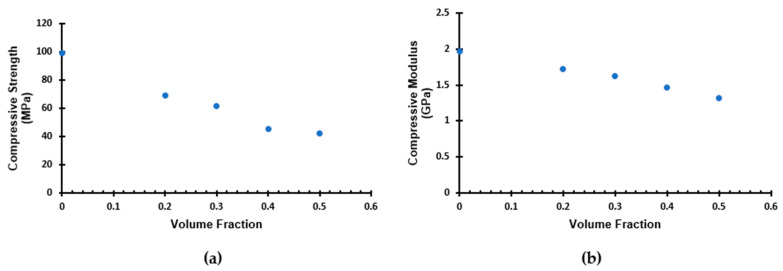
Effect of the microballoon volume fraction on (**a**) compressive strength; (**b**) compressive modulus.

Furthermore, Table 2 allows for the analysis of the effect of the volume fraction of the microballoons on the mechanical characteristics of the syntactic foams. As the microballoon volume fraction rises, the average compressive strength of the foam gradually declines, as shown in Figure 5a. Additionally, the specimens’ compressive moduli also show the effect of the volume fraction (Table 2 and Figure 5b). The trend is similar to that of the average compressive strength, where the average compressive modulus of the foam decreased as the volume fraction of the glass microballoon in the syntactic foam increased. The average compressive strength of the foams decreased by 29.85% to 57.02% in comparison to the neat resin, while the foams’ compressive modulus decreased by 12.76% to 32.84%. These trends directly relate to the amount of resin present in the syntactic foam. When resin content is high in the syntactic foam, the resin acts as the load-bearing phase. However, with higher content of microballoons, they start to take up fewer loads under compression. Since the thin-walled, low-density K25 microballoons have a poor load-bearing capacity, the compressive strength of the syntactic foam as a whole decreases. This trend can also be attributed to the fact that the void content on the syntactic foam with higher microballoon volume fraction foam was comparatively greater than those with lower microballoon volume fractions.

**Figure 6:**
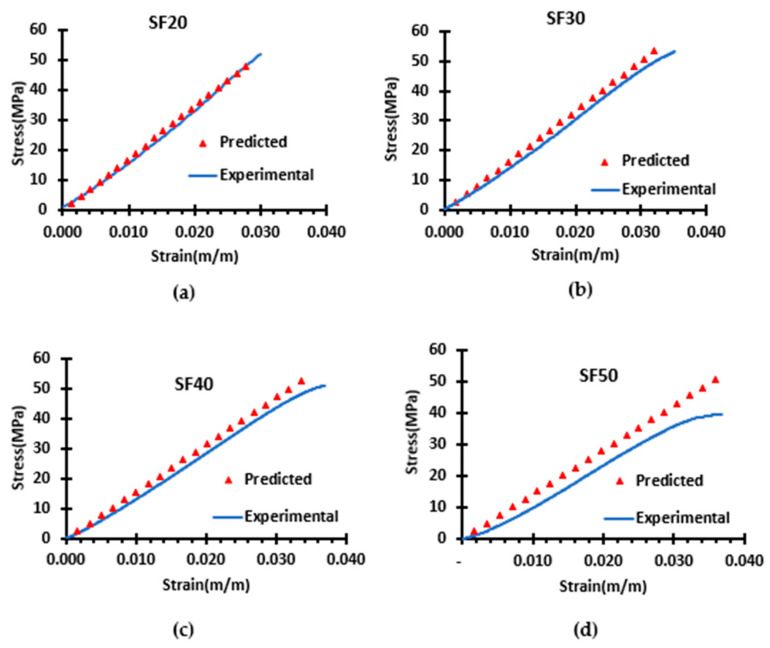
Comparison of the measured and predicted stress–strain curves in the elastic region of syntactic foam (**a**) SF20, (**b**) SF30, (**c**) SF40, and (**d**) SF50.

The finite element simulation of the 3D model was also used to study the elastic region of the syntactic foams. As illustrated in Figure 6, the predicted stress–strain curves in the elastic region of the foams closely match those obtained from the compression test of the syntactic foam. The consistency of the curves supports the simulation model’s validity. Furthermore, the predicted compressive modulus is less than 10% higher than the measured one, as shown in Figure 5b. This inconsistency in the results can be attributed to the approximation in the geometry of the simulation model, as the 3D model used in the simulation does not consist of any defects that are usually present in the experimental specimen like air bubble voids. This also explains why the model data and experimental data diverge more in the higher SF50 compared to the SF20, as the void content is much higher in SF50 as compared to SF20.

**Figure 7:**
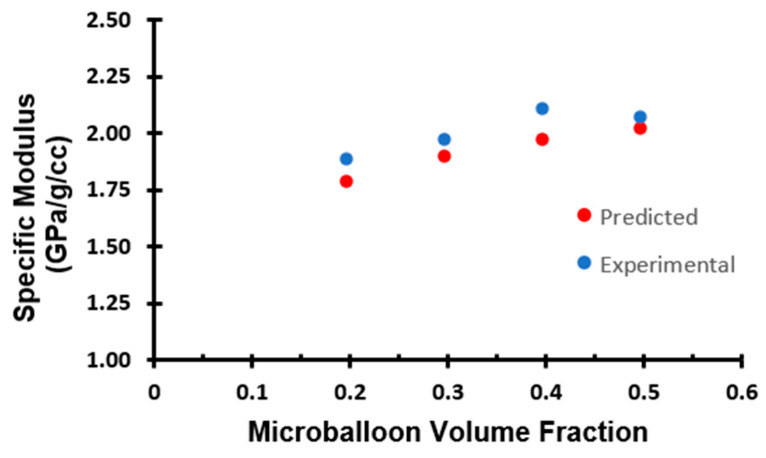
Comparison of the effect of the microballoon volume fraction on measured and predicted specific modulus.

Cracks could easily initiate locally in the presence of a void or other defects. As a result, the foam containing a higher volume fraction of microballoons could fail more easily. In contrast, comparing the specific modulus of all the specimens, the trend is different than that of the compressive strength and the compressive modulus. As the microballoon volume percentage increases, as shown in Table 2 and Figure 7, the specific modulus of syntactic foam gradually increases. This indicates that, when the volume percentage of the microballoon increases, the strength-to-weight ratio of the foam also increases. This is due to the reduction in foam density with the addition of microballoons [9].

### 3.3. Failure Mechanism

**Figure 8:**
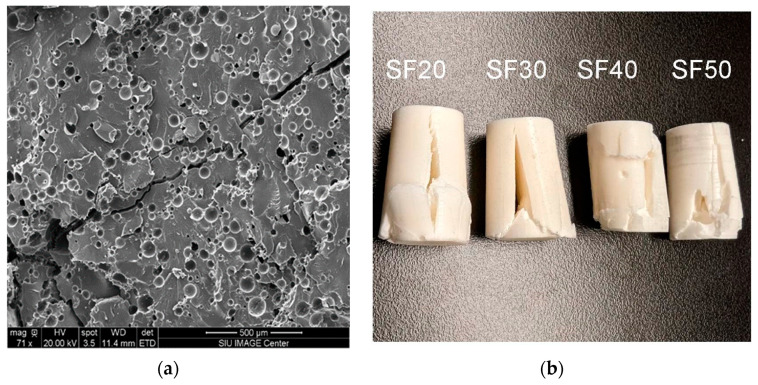
(**a**) SEM image of the failed syntactic foam sample SF20 (taken precisely in the middle of the failed sample) and (**b**) failed SF20, SF30, SF40, and SF50 samples showing the macro-crack.

The rupture of the microballoons and the development of microcracks in the epoxy matrix are the root causes of syntactic foam failure. These microcracks and microballoon fractures increase when additional load is applied to the samples, merge to form a macrocrack, and ultimately lead to sample failure. This is apparent in the SEM micrograph in Figure 8a, where a diagonal macro-crack is formed by the crack primarily extending through the matrix that adjoins all the smaller cracks. This also helps to explain the lack of a significant plateau region during compression testing, as discussed in Section 3.2. The failed samples shown in Figure 8b display that the dominant cracking path in these samples is longitudinal regardless of the microballoon content. This suggests that the microballoon volume percentage has no discernible impact on the way that foams fail. The longitudinal cracks also suggest that the crack propagation in the foam mainly occurs in the matrix [19].

### 3.4. Viscoelastic Properties

**Figure 9:**
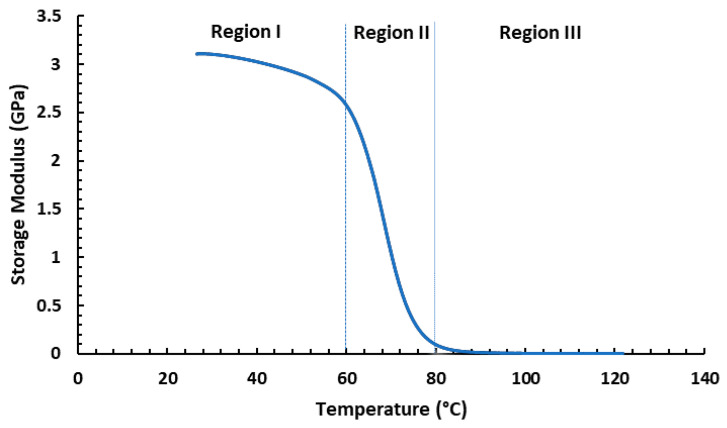
Storage modulus curve showing different regions.

Storage modulus plots for syntactic foams can be divided into three separate zones, as shown in Figure 9 [24]. At Region I, the material’s storage modulus gradually lowers as the temperature rises; at Region II, the storage modulus temperature sharply drops with rising temperature located near the transition temperature (Tg); and finally, in Region III, a rise in temperature causes the storage modulus to stabilize at a very low value compared to Region I.

**Figure 10:**
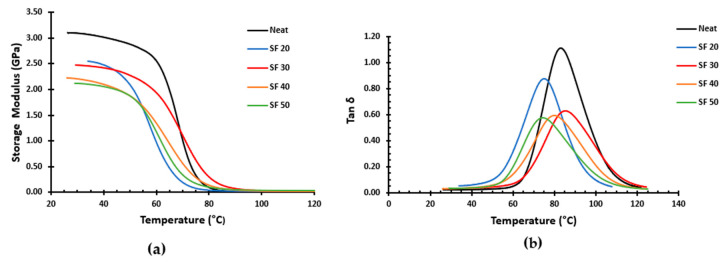
DMA results of syntactic foams and neat resin showing (**a**) storage modulus curve, (**b**) tan δ curve.

Figure 10a depicts the representative collection of graphs for the storage modulus change in several types of syntactic foam and neat resin with respect to temperature. We investigated the storage modulus value at two representative temperatures of 35 °C and 105 °C, as given in Table 3, to further explain the impact of the volume fraction of microballoon on the syntactic foam storage modulus. A majority of the application of the syntactic foams takes place at 35 °C, which is in Region I of the graph and near room temperature. At this temperature, an increase in the microballoons volume fraction of syntactic foam causes the storage modulus of the material to decrease; 105 °C is located in Region III of the graph, where the storage modulus of syntactic foams tends to stabilize. These trends can be due to the softening of the matrix. The matrix is stiff at room temperature, which causes a greater value for the syntactic foam’s storage modulus. Hence, the foam with the least volume of the matrix, SF50, has the lowest storage modulus. After the transition temperature, the matrix starts to become softened, but since the glass microballoons do not have the same transition temperature, the rigid microballoons contribute towards the stiffness of the foam. Hence the foam with the maximum volume of microballoon, SF50, has the highest storage modulus at Region III after the transition temperature of the foams. In addition, as shown in Figure 10a, when comparing the neat resin’s storage modulus graph to that of all the foams, the neat resin’s storage modulus is highest in Region I and lowest in Region III.

The graph in Figure 10b depicts the variance in the tan delta (tan δ) values for neat resin and syntactic foams of different volume percentages. The values of the tan δ for each sample of the syntactic foam and the neat resin are similarly listed in Table 4 for 35 °C and the maximum. The tan δ values at 35 °C are not considerably different; however, the maximum tan δ values of the foams are much lower than those of the neat resin. The components of a composite and their interactions with one another affect its damping properties. The rigid glass microballoon impairs the material’s elasticity and reduces the foam’s ability to dissipate energy. Therefore, we can see the decreasing trend of the tan δ_max_ values in the specimens as there is an increase in microballoon content.

## 4. Conclusions

The effect of the microballoon volume fractions on the elastic and viscoelastic properties of the syntactic foam was studied. Both 3D modeling and experimental methods were utilized to characterize the foams containing varying K25 microballoons volume fractions (0.2 to 0.5). Void content in the foams increased with the increase in the volume fraction of microballoons. A gradual decrease in the average compressive strength and the compressive modulus of the foam with an increase in the microballoon volume fraction was observed. The inclusion of the microballoons reduced the resin’s compressive strength by up to 57.017% and its compressive modulus by up to 32.841%. On the contrary, the specific modulus increased due to the addition of a low-density microballoon which reduced the density of syntactic foam. The storage modulus of the syntactic foam gradually decreased with an increase in the volume fraction of the microballoons. However, it gradually improved with the increase in the microballoon volume fraction at higher temperatures. After the softening of the matrix, the rigid microballoons provided the reinforcement, resulting in the higher storage modulus values of syntactic foams compared to the neat resin. Similarly, the presence of the rigid glass microballoon also affected the flexibility of the material and lowered the energy dissipation in the foam which caused the decreasing trend of the maximum tan δ values of foams compared to the neat resin. This study was focused on only one type of matrix and microballoons. As a result, the generalization of the relationship between the elastic and viscoelastic properties and the volume fraction of the microballoons in the syntactic foam cannot be confirmed. In the future, a greater range of material constituents should be tested and analyzed both numerically and experimentally to establish a generalized relationship between the microballoon volume fraction and the elastic and viscoelastic properties. The numerical method in this study addressed the elastic region of the stress–strain curve with the static analysis of the 3D model. More advanced simulation work can be carried out to predict both the linear and nonlinear behavior of the foams under both static and dynamic loading.

## Figures and Tables

**Table 1:** Composition, density, and void content of the syntactic foams.

Samples	Microballoon Volume (%)	Average Density (g/cc)	Theoretical Density (g/cc)	Void Content (%)
Neat	0	1.145 ± 0.003	1.160	1.276 ± 0.235
SF20	20	0.967 ± 0.002	0.978	1.151 ± 0.173
SF30	30	0.861 ± 0.002	0.887	2.970 ± 0.272
SF40	40	0.744 ± 0.010	0.794	6.242 ± 1.307
SF50	50	0.657 ± 0.002	0.702	6.393 ± 0.339

**Table 2:** Average compressive modulus, compressive strength, and specific modulus of the syntactic foams.

Samples	Average Compressive Modulus (GPa)	Average Compressive Strength (MPa)	Specific Modulus (GPa/(g/cc))
Neat	1.970 ± 0.007	98.832 ± 4.252	1.662 ± 0.016
SF20	1.718 ± 0.073	69.335 ± 3.520	1.777 ± 0.075
SF30	1.627 ± 0.023	61.428 ± 2.791	1.891 ± 0.031
SF40	1.459 ± 0.093	45.105 ± 5.773	1.962 ± 0.146
SF50	1.323 ± 0.100	42.481 ± 2.614	2.012 ± 0.156

**Table 3:** Comparison of storage modulus E′, transition temperature Tg, and loss modulus E″.

Samples	E′ at 35 °C (GPa)	E′ at 105 °C (GPa)	T_g_	E″ at 35 °C (GPa)
Neat	3.067	0.004	66.62	0.062
SF20	2.536	0.011	57.30	0.135
SF30	2.457	0.024	67.18	0.087
SF40	2.170	0.026	61.72	0.078
SF50	2.088	0.036	57.71	0.068

**Table 4:** Comparison of tan δ values at 35 °C and maximum tan delta values.

Samples	Tan δ at 35 °C	Tan δ_max_
Neat	0.020	1.108
SF20	0.053	0.875
SF30	0.035	0.625
SF40	0.036	0.591
SF50	0.032	0.573

## Data Availability

The data presented in this study are available on reasonable request from the corresponding author.
